# Perceived visual time depends on motor preparation and direction of hand movements

**DOI:** 10.1038/srep27947

**Published:** 2016-06-10

**Authors:** Alice Tomassini, Maria Concetta Morrone

**Affiliations:** 1 Department of Robotics, Brain and Cognitive Sciences, Istituto Italiano di Tecnologia, Via Morego 30, 16163 Genova, Italy; 2 Donders Institute for Brain, Cognition, and Behaviour, Centre for Cognition, Radboud University Nijmegen, 6525 HR Nijmegen, The Netherlands; 3 Department of Translational Research on New Technologies in Medicine and Surgery, University of Pisa, via San Zeno 31, 56123 Pisa, Italy; 4 Scientific Institute Stella Maris, Viale del Tirreno 331, 56018 Calambrone, Pisa, Italy

## Abstract

Perceived time undergoes distortions when we prepare and perform movements, showing compression and/or expansion for visual, tactile and auditory stimuli. However, the actual motor system contribution to these time distortions is far from clear. In this study we investigated visual time perception during preparation of isometric contractions and real movements of the hand in two different directions (right/left). Comparable modulations of visual event-timing are found in the isometric and in the movement condition, excluding explanations based on movement-induced sensory masking or attenuation. Most importantly, and surprisingly, visual time depends on the movement direction, being expanded for hand movements pointing away from the body and compressed in the other direction. Furthermore, the effect of movement direction is not constant, but rather undergoes non-monotonic modulations in the brief moments preceding movement initiation. Our findings indicate that time distortions are strongly linked to the motor system, and they may be unavoidable consequences of the mechanisms subserving sensory-motor integration.

Our brain flexibly deals with multiple, often misaligned, times, despite the strong perception of a unitary time. Perceived time can differ across sensory modalities, features of the sensory stimulation and even positions in space^1^,^2^,^3^,^4^,^5^. To account for this inherent adaptability, the classical idea of a dedicated clock is giving way to the notion that time might arise from the intrinsic dynamics of local neuronal activity^6^,^7^,^8^.

Intriguingly, the motor system appears to have a key role in shaping perceived time. Multifaceted effects, including compression and dilation of time, have been reported in different epochs surrounding action execution^9^,^10^,^11^,^12^,^13^,^14^,^15^,^16^,^17^,^18^. Some of these misperceptions of time show striking similarities across the different sensory-motor systems. The temporal separation between a pair of visual flashes is remarkably shortened around the time of saccadic eye movements so that the flashes may even appear with inverted order^11^,^19^. A similar tactile event order inversion has been associated with the crossing of the hands^20^ and tactile temporal compression, analogous to the perisaccadic visual time perception, has been reported shortly before the execution of a hand movement^14^. Notably, the compression of tactile time is not widespread to all body locations, but rather is confined within the hand effector, indicating the involvement of selective sensory-motor processes.

For both the visual and the haptic system these changes in perceived time are paralleled by concomitant changes in perceived space, mainly consisting in mislocalizing visual and tactile stimuli towards the target of an eye or hand movement, respectively^21^,^22^,^23^. The alterations in space-time perception begin prior to the actual movement onset (up to ~200 ms; see^14^)-during the motor preparatory stage-and unfold with similar temporal dynamics locked to movement execution^19^. This suggests not only that time and space are likely interlaced in the brain, but their joint modulation might be an integral part of basic sensory-motor functions. Indeed, one account posits that these phenomena are consequences of a complex mechanism, primarily driven by motor signals, that aims at integrating sensory information across movements^24^,^25^. However, alternative explanations have been put forth claiming that perisaccadic misperceptions of time (and space) are neither inherent nor exclusive to sensorimotor processing but rather represent accidental effects due to movement-induced visual masking or attenuation^26^,^27^,^28^.

Interestingly, action-related temporal distortions are not restricted to the sensory channel that is directly linked to the relevant motor system (i.e., vision for the oculomotor system or touch for the haptic system). Perisaccadic reversal of subjective time has been documented not just for visual but also for tactile stimuli^29^. Conversely, hand movements can affect visual time. Yokosaka and colleagues^18^ recently reported velocity-dependent compression of visual intervals during the execution of hand movements. Temporal expansion, on the contrary, has been observed for visual stimuli presented during preparation for reaching movements^13^ and the effect is modulated by the degree of motor preparation. Most interestingly, it is accompanied–and possibly explained-by changes in visual information processing, as indicated by the concomitant improvement in detection performance for rapidly displayed visual targets^13^. The available evidence for supra-modal effects suggests that the time distortions might be linked to the motor system and not to the specific sensory system involved. Yet, whether motor signals play a fundamental role in their establishment has still to be demonstrated.

It has been shown that these perceptual effects emerge already before movement and can be of opposite sign–i.e., compressions and expansions of time–probably depending on the specific phase of the motor task under investigation, for example motor execution or preparation. Furthermore, as suggested by Hagura and colleagues (2012), they might result from more general changes in sensory processing occurring at the time of movement which may be inherent to sensory-motor coupling functions.

If time distortions are driven by early motor signals during the preparatory stage we might expect modulations by specific features of the planned movements, even before movements are actually implemented. This study addresses this hypothesis by investigating the time course of visual temporal distortions during preparation for hand movements and its specificity for different movement directions (right/left). To isolate the contribution of motor planning processes and rule out any confound due to sensory masking or spatial effects induced by hand displacement we tested both an isometric contraction and a real movement condition.

## Methods

### Apparatus

Participants sat in front of a table in a completely dark room with their right hand resting on a hand-shaped plaster cast. The plaster cast was mounted on a sliding guide (length 1.5 m) that constrained hand movement along one single direction. Forces and torques produced by the hand were measured by means of a six-axis force/torque transducer (Mini40 F/T transducer; ATI Industrial Automation) fixed to the lower surface of the hand plaster cast.

The fixation point and the visual stimuli were provided by a red (diameter 0.5 cm) and a yellow (diameter 1 cm) light-emitting diode (LED), respectively. Auditory tones were presented with two loudspeakers placed on the table, ~10 cm from each other and ~40 cm from the participant. The subjects could not see their hand or any other light or visual feature except the two LEDs when lighted (5 ms).

The LEDs were operated by means of a National Instrument data acquisition board (NI-USB 6211; sampling rate 1000 Hz) controlled via a custom-made Matlab program allowing a fine control of stimulus presentation timing.

The voltage signals from the force sensor and the LEDs were all acquired with the same data acquisition board (sampling rate 1000 Hz) and accurately aligned in time. For the *isometric contraction* condition, but not for the *movement* condition (see below for explanations about the experimental conditions), the voltage signal from the loudspeakers was also acquired with the same acquisition board allowing precise estimates of the reaction times.

### Procedure

**Figure 1:**
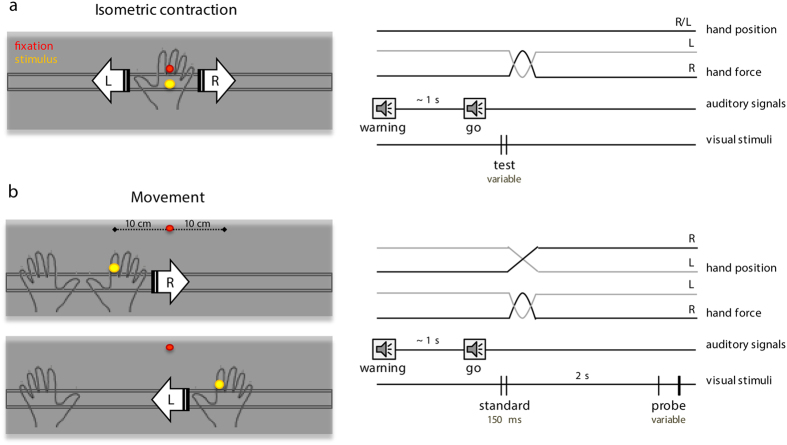
Schematic illustration of the experimental setup and procedure. (**a**) Isometric contraction condition. Participants kept their right hand on a hand-shaped plaster cast that was mounted on a sliding guide. Two LEDs were positioned above the hand: the red LED was the fixation point and the yellow LED delivered the visual stimuli (left panel). An auditory tone (warning signal) marked the beginning of the trial. After a variable delay, a second tone (go signal) cued participants to push their right hand either to the right or to the left direction according to previous verbal instructions. The hand plaster cast was securely blocked so that the horizontal force did not result in hand displacement. At random times with respect to the go signal, two visual flashes (5 ms) were presented marking a variable time interval– the test interval (right panel). At the end of the trial participants were asked to tell whether the test was shorter or longer compared with the standard interval of 150 ms (shown 10 times consecutively at the beginning of each block of trials). (**b**) Movement condition. The red fixation LED was attached on the table, above the sliding guide; the yellow LED was mounted on the right index finger. The hand plaster cast was let free to slide along the guide. Participants positioned their right hand ~10 cm to the left and ~10 cm to the right with respect to the body midline prior to executing a leftward or a rightward movement, respectively (left panel). Like in the isometric condition, two auditory tones were presented; the second tone signaled participants to move their hand ~20 cm to the right or left direction, according to previous instructions. Two time intervals were presented: the standard-150 ms-was delivered at random times with respect to the go signal; the probe, of variable duration, was delivered 2 s after the standard, when the hand was still (right panel). Participants indicated verbally at the end of the trial which of the two visual intervals was longer.

Participants were asked to judge the temporal separation between two visual flashes while performing a motor task with their right hand. We tested two different conditions where the motor task consisted either in contracting (*isometric contraction* condition) or moving (*movement* condition) the hand towards the right and left direction (Fig. 1). Rightward and leftward hand movements were randomly intermingled within each block of trials.

In the *isometric contraction* condition (Fig. 1a) the fixation and the stimulus LED were positioned above the right hand with a Velcro strap, 3.5 cm from each other (fixation LED top; stimulus LED bottom) observed at a distance of ~40 cm. Data were collected in separate blocks of ~45 test trials each. At the beginning of each block of trials participants were presented with 10 repetitions of the standard stimulus delivered by the yellow LED. They were encouraged to pay attention to the standard interval and to try to remember it for future comparisons. The standard stimulus was an empty temporal interval of 150 ms marked by two brief visual flashes of 5 ms each. Each repetition of the standard interval was separated by a random pause drawn from a uniform distribution ranging from 0.5 to 0.8 s. After the sequence of standards was completed the test phase began. Before each trial the experimenter gave a verbal instruction about whether the participants had to push their hand to the right or to the left just as if they intended to move their hand along the sliding guide. The fixation LED turned on and 0.5 s later an auditory tone (900 Hz; 30 ms) marked the beginning of the trial. After a variable delay ranging from 0.7 to 1.3 s a second, identical tone (go signal) cued participants to push their right hand as soon as possible either to the right or to the left according to the previous instruction. The hand plaster cast was securely blocked so that the horizontal force did not result in hand displacement. At random times with respect to the go cue, the test interval was presented and participants were asked to tell at the end of the trial whether it was shorter or longer compared with the standard interval (shown at the beginning of the block). The duration of the test interval varied randomly on a trial-by trial basis from ~60 to ~240 ms in steps of 10 ms (although the exact range of stimulus variation and its grain was slightly adjusted by the experimenter according to the subject’s performance). Stimulus latencies varied randomly within a ~400 ms range centered around the go signal presentation time so as to maximize sampling in the temporal window just before hand force onset time. An automatic algorithm adjusted stimulus latencies during each block of trials according to the participant’s reaction times.

In the *movement* condition (Fig. 1b) the fixation LED was attached on the table 12 cm from the sliding guide and approximately halfway between the starting and final hand position on the horizontal plane (see below). The stimulus LED was mounted on the right index finger with a Velcro strap. Like for the *isometric contraction* condition, the experimenter told the participant at the beginning of each trial whether to perform a rightward or a leftward movement. The starting hand position changed depending on the direction of the movement to be performed, being ~10 cm to the left and ~10 cm to the right with respect to the body midline for rightward and leftward movements, respectively. The participants positioned their hand in the correct starting position prior to the beginning of each trial, as soon as the experimenter gave the verbal instruction about the next movement direction. Once the hand was in the correct position, the fixation LED was lighted up (and stayed on for the entire trial duration) and the two successive auditory tones were presented as previously described for the *isometric contraction* condition. Participants had to move their hand ~20 cm to the right (or to the left) as soon as possible after the go signal.

Two pairs of visual flashes (5 ms each) were presented marking two empty temporal intervals. The first interval –the standard- had fixed duration of 150 ms and was delivered at random times with respect to the go signal so as to maximize stimulus sampling just before movement onset time in the same way as already described for the *isometric contraction* condition. 2 s after the presentation of the standard interval, when the movement was over and the hand was completely still, the second interval was delivered–the probe- with a variable duration (from ~60 to ~240 ms in steps of 10 ms). Participants indicated verbally at the end of the trial which of the two intervals was longer. Data were collected in separate blocks of ~60 trials each.

Before beginning the experiment participants underwent a preliminary training phase (~50 trials) to learn how to execute the instructed hand contractions and movements (~500 ms in duration) in response to the go signal. This familiarization phase provided also an estimate of the precision of participants in the temporal discrimination task that was subsequently used to set the initial width and grain of the range of stimuli used in the experiment. The practice trials were also used to evaluate the subject’s mean reaction time to appropriately set the initial stimulus presentation times so that sampling was mostly concentrated in a ~300 ms temporal window before force onset time.

Five naïve participants (three females, age = 28 ± 1 SD years) were tested in the *isometric contraction* condition. Each participant completed at least 26 blocks of trials in different testing days (mean number of trials per participant 1233 ± 52). Two naïve participants (one female; 24 and 32 years) were tested in the *movement* condition. One participant completed 14 and the other 12 blocks of trials in two different testing days (mean number of trials per participant 746 ± 101). All participants provided written informed consent prior to testing. The study was approved by the local ethics committee (Azienda Sanitaria Locale Genovese N.3) and the experimental procedure was conducted in accordance with the approved guidelines and rules.

### Data Analysis

Data analysis was performed off-line. Force onset time was determined by an automated algorithm as the instant corresponding to the first sample of a series of 15 consecutive samples (15 ms with a 1000 Hz sampling rate) where the first derivative of the force was greater than zero. This computation was applied to the force component that was aligned to the direction along which the participants pushed (i.e., along the axis parallel to the direction of the hand contraction/movement). Test presentation times were then expressed relative to the central point of the temporal interval marked by the test pair. Stimulus latencies were calculated as the difference between test presentation time and force onset time. Thus, negative and positive latency values indicate that the center of the test interval fell before and after the onset of hand contraction/movement, respectively.

Data from each participant were binned (bin size = 50 ms) according to stimulus latency and then fitted separately with cumulative Gaussian functions, estimated by means of the Maximum Likelihood method. Bin size was chosen so that psychometric functions were never fitted to datasets with less than 25 trials. 84.6 ± 4.5 (SE) trials served on average to calculate each psychometric function. Both the Point of Subjective Equality (PSE) and the Just Noticeable Difference threshold [corresponding to the standard deviation (SD) of the fitted cumulative Gaussian function] were derived from the psychometric function parameters. The standard errors (SEs) of the PSEs and SDs were estimated by bootstrap.

#### Group analysis

PSEs and SDs for the isometric contraction condition were subjected to a two-way repeated-measures ANOVA analysis with direction (right, left) and latency (−0.175, −0.125, −0.075, −0.025, + 0.025 s) as within-subject factors. One-tailed post-hoc t-tests were run to determine the time points (relative to force onset) where perceived duration for the right direction was significantly expanded with respect to the left direction.

Data from the isometric contraction condition (n = 5) have been combined with the data collected in the real movement condition (n = 2) and submitted to a joint two-way repeated measures ANOVA (n = 7). Since we did not collect data at −0.175 s in the real movement condition, when combining the data from the two movement conditions we considered only the latencies from −0.125 to + 0.025 s. The ANOVA for the combined data set was run for four levels of factor latency (−0.125, −0.075, −0.025, +0.025 s) instead of five levels. Post-hoc tests were again run to determine the time points where perceived time was significantly different between the right and left direction.

#### Single-subject analysis

To test for the statistical significance at the single-subject level, the individual data were subjected to a permutation test^30^ by shuffling the left and the right labels, while maintaining the correct stimulus interval and latency labels at two critical time points preceding movement onset (−0.075 and −0.025 s). We randomly permuted trials across the right and left conditions with 1000 iterations for each subject. We fitted a psychometric curve and estimated the PSE for each random partition and the difference between the obtained PSEs. This procedure generates an estimate of the distribution of the difference between the PSEs under the null hypothesis that the data for the right and left direction derive from the same probability distribution (i.e., the probability distributions for the right and left conditions are exchangeable). We then calculated the proportion of random permutations that resulted in a larger difference in the PSE than the observed difference between the right and left direction. This proportion represents the p-value.

## Results

**Figure 2:**
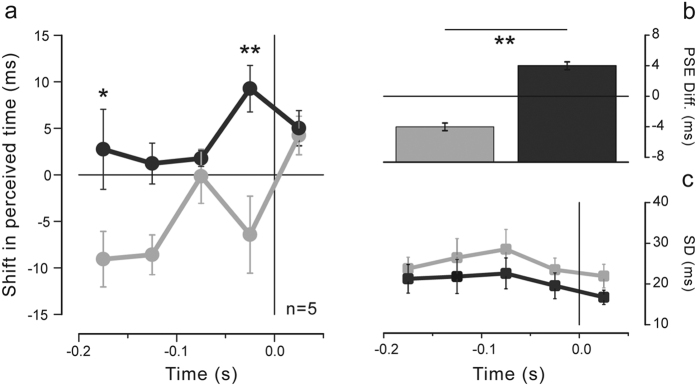
(**a**) Perceived time as a function of stimulus presentation time with respect to movement onset time (zero time) averaged across subjects for the right (black) and left (gray) hand force direction in the isometric contraction condition. Results are expressed as the deviation of the PSEs from the mean individual PSE (i.e., the PSE averaged across stimulus latencies and movement directions, calculated separately for each subject). Negative and positive values indicate relative underestimation and overestimation of the time interval, respectively. Asterisks represent the significance level of one-tailed post-hoc t-tests (*p < 0.05, **p < 0.01; Bonferroni-corrected for multiple comparisons). (**b**) Average deviation in the perceived temporal interval for the right (black) and left (gray) hand force directions. Asterisks represent the significance level of the paired-samples t-test. (**c**) Precision of the temporal judgments (SDs) as a function of stimulus presentation time with respect to movement onset time averaged across subjects for the right (black) and left (gray) hand force direction. Error bars represent standard errors of the means.

The time course of the perceived visual temporal separation in the isometric contraction condition aligned to force onset is shown in Fig. 2a for the right (black) and left (gray) force directions (average results, n = 5). Negative and positive shifts indicate, respectively, temporal underestimation and overestimation relative to the mean perceived interval (i.e., the PSE averaged across stimulus latencies and force directions calculated for each subject separately). The perceived time varies in a complex, non-monotonic fashion depending on both stimulus latency as well as hand force direction. Visual temporal intervals are underestimated when preparing to move the right hand to the left compared to the right direction. The systematic difference in temporal judgments between the two opposite force directions is highly consistent across participants (t_(4)_ = −7.958, p = 0.001, paired samples two-tailed t-test), though rather small, being on average 8 ± 1 ms (MEAN ± SE; see bars in Fig. 2b). Indeed, the difference in perceived time for the two hand directions is not constant during motor preparation but changes as a function of stimulus latency relative to force onset time. Well before (~−0.15 s) and shortly before (−0.025 s) muscle contraction, perceived time shows maximal separation between the two hand force directions. This difference, however, almost vanishes at intermediate times, approximately around −0.075 s as well as when hand contraction has been just initiated (+0.025 s). A two-way repeated-measures ANOVA on the PSEs with direction (right, left) and latency (−0.175, −0.125, −0.075, −0.025, +0.025 s) as within-subject factors shows that temporal intervals are perceived as shorter for the left compared to the right direction (F_(1,4)_ = 63.335, p = 0.001; main effect of factor direction) with no systematic effect of stimulus latency (F_(4,16)_ = 1.795, p = 0.179; main effect of factor latency). Importantly, this analysis yields a significant interaction between factor direction and latency (F_(4,16)_ = 3.843, p = 0.023) confirming that perceived time is modulated in a different way during preparation to move the hand to the right and to the left.

**Figure 3:**
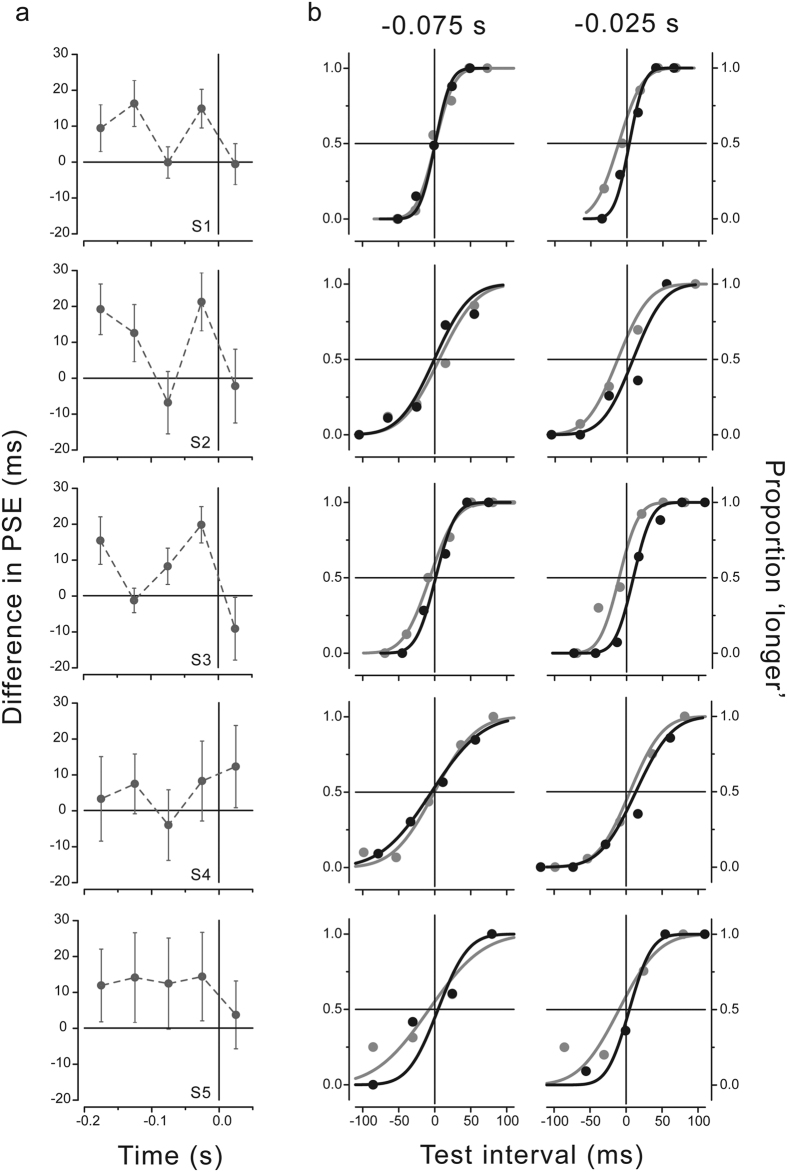
(**a**) Difference between the PSEs for the right and left hand force direction as a function of stimulus presentation time with respect to movement onset time (zero time). Results for all subjects. Error bars represent standard errors estimated by bootstrap. (**b**) Psychometric functions showing the proportion of trials where the test stimulus interval was judged to be longer than the standard interval in the isometric condition. Data for all subjects are shown for two critical stimulus presentation times relative to force onset (−0.075 and −0.025 s; bin size of 50 ms) for the right (black) and left (gray) movement directions. The test stimulus interval is expressed as deviation from the mean individual PSE. The vertical and horizontal lines represent no deviation from the mean perceived time.

Figure 3a highlights the fluctuating pattern in perceived time by showing the difference in the PSEs between the right and left directions (results for all participants between −0.175 and +0.025 s). The difference between the two directions is not constant but rather shows similar discontinuities in almost all subjects (except S5), suggesting that perceived time may undergo opposite changes during preparation for a right (relative time expansion) and left (relative time compression) hand movement. One-tailed post-hoc t-tests corroborate the alternating pattern of the temporal effects showing that perceived time for the right direction is significantly expanded with respect to the left direction at −0.175 s (mean difference = 11.8 ± 2 ms; p = 0.03, Bonferroni-corrected) as well as at −0.025 s (mean difference = 15.7 ± 2 ms; p = 0.005, Bonferroni-corrected), but not at interwoven latencies, such as −0.125 s (mean difference = 9.8 ± 3 ms; p = 0.09, Bonferroni-corrected), −0.075 s (mean difference = 1.9 ± 4 ms; p = 0.626) and + 0.025 s (mean difference = 0.8 ± 3 ms, p = 0.836).

The psychometric functions fitted to the data at two critical latencies just preceding force onset (−0.075 and −0.025 s) are shown for all participants in Fig. 3b. The fitted curves are superimposed at −0.075 s while they diverge at −0.025 s before force onset. This pattern of results is shown by all participants, though the differences in the PSEs at −0.025 s are stronger and statistically significant at the individual subject level for S1 (p = 0.002), S2 (p = 0.009) and S3 (p < 0.001; see single-subject analysis in the Methods section). In agreement with the post-hoc tests, none of the participants shows a significant difference in the PSEs at −0.075 s.

A different pattern of results is observed for the precision of the temporal judgments (SD; Fig. 2c) that shows only a slight, yet non-significant, tendency to be higher when preparing to move to the right with respect to the left direction (F_(1,4)_ = 1.790, p = 0.252; main effect of factor direction). No significant effect of stimulus latency (F_(4,16)_ = 2.429, p = 0.09) as well as no significant interaction between direction and latency (F_(4,16)_ = 0.16, p = 0.955) are also reported, indicating that both movement preparation and movement direction do not affect in any way the precision of the temporal estimates.

**Figure 4:**
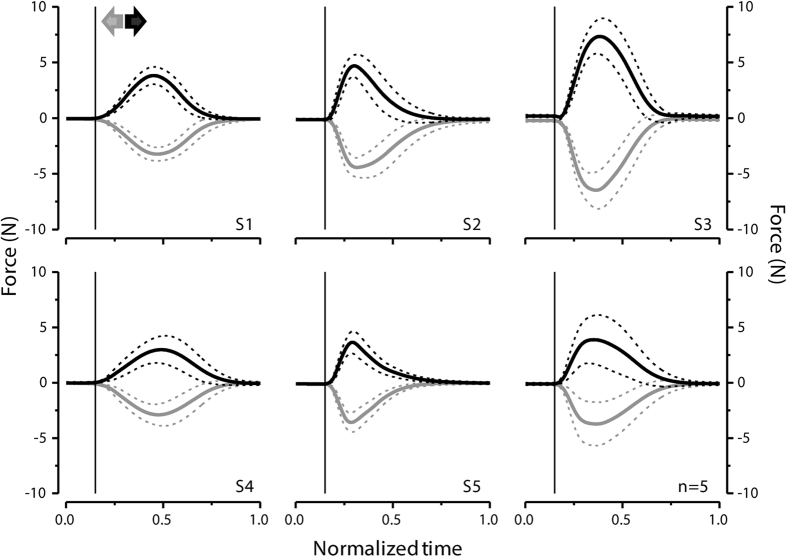
Force profiles for the right (black) and left (gray) directions. Forces have been aligned to force onset time and then averaged across trials for each subject (thick lines). Dashed lines represent MEANS ± 1SD. Vertical lines indicate hand force onset time. Individual and mean (n = 5; right bottom graph) force profiles are shown.

Motor performance is comparable for the rightward and leftward movements. Reaction times are not significantly different when pushing the hand to the right and left direction (right = 205 ± 7 ms; left = 206 ± 10 ms; t_(4)_ = 0.124, p = 0.908; two-tailed paired-samples t-test), indicating that the motor task was equally demanding in the two conditions. Force profiles for both directions are bell-shaped with comparable peak forces (right = 5.1 ± 0.8 N; left = 4.6 ± 0.7 N; t_(4)_ = −2.18, p = 0.095; two-tailed paired-samples t-test) and times-to-peak (right = 234 ± 39 ms; left = 233 ± 39 ms; t_(4)_ = −0.126, p = 0.906; two-tailed paired-samples t-test; see Fig. 4). Overall, these results show that no other basic feature of the motor performance, aside from direction, is likely to play a role in the observed modulations of temporal judgments for rightward and leftward movements.

**Figure 5:**
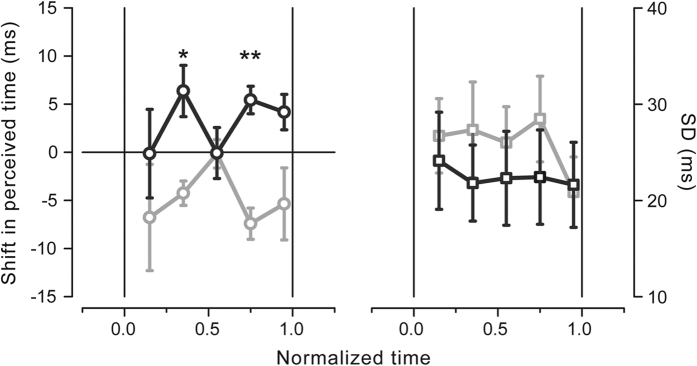
Time course of the shift in perceived time when stimulus latencies are normalized on the reaction time on a trial-by-trial basis for the right and left hand directions (results are averaged across subjects; left graph). Time 0 represents the go signal presentation time; time 1 represents the hand force onset time. Mean precision of the temporal judgments (SDs) for the data normalized on the reaction times are shown in the right graph. Error bars represent standard errors of the mean. Asterisks represent the significance level of one-tailed post-hoc t-tests (*p < 0.05, **p < 0.01; Bonferroni-corrected for multiple comparisons).

To investigate the temporal dynamics of the observed modulations in more detail we normalized stimulus latencies on the individual reaction times (on a trial-by-trial basis). This normalization procedure preserves the anti-phase pattern in the temporal judgments and shows that perceived time becomes virtually identical for the two hand directions approximately mid-way between the go signal presentation (zero time) and hand force onset (time 1), that is at half of the reaction time interval (Fig. 5, left graph). Just before and just after this central point perceived time undergoes opposite modulations, compressing when preparing to move left and expanding when preparing to move right; higher inter-subject variability in the temporal judgments is instead observed for the earliest and the latest time point. Confirming the results found for the data aligned to force onset time, the data from the normalized times 0.35, 0.55 and 0.75 show a significant statistical interaction between direction and latency (F_(2,8)_ = 4.902, p = 0.041; two-way repeated measures ANOVA) and a significant effect of movement direction (F_(1,4)_ = 33.936, p = 0.004).

Post-hoc one-tailed t-tests indicate relative expansion of perceived time for the right compared to the left direction at 0.35 (p = 0.048, Bonferroni-corrected) and at 0.75 (p = 0.012, Bonferroni-corrected) but not half-way of the reaction time interval (p = 0.979).

Again, no significant modulations in precision are found after normalizing stimulus latencies on the individual reaction times (F_(1,4)_ = 2.207, p = 0.212; main effect of direction; F_(2,8)_ = 0.928, p = 0.434; main effect of latency; F_(2,8)_ = 0.157, p = 0.858; interaction effect; Fig. 5, right graph).

**Figure 6:**
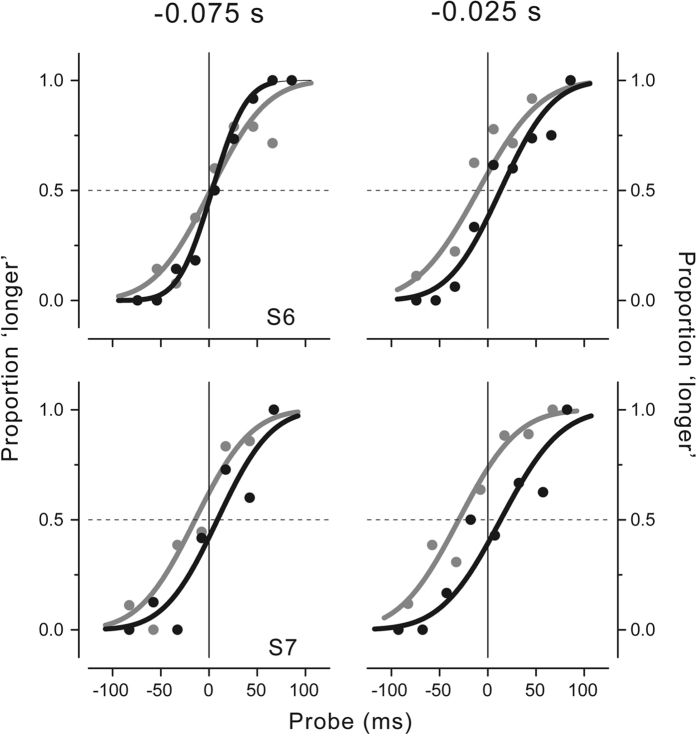
Psychometric functions showing the proportion of trials where the probe was judged to be longer than the standard interval in the movement condition. Data for the two tested subjects are shown for two critical stimulus presentation times relative to movement onset (−0.075 and −0.025 s; bin size of 50 ms) for the right (black) and left (gray) movement directions. The probe is expressed as deviation from the mean individual PSE (i.e., the PSE averaged across stimulus latencies and movement directions, calculated separately for each subject; S6 = 154 ± 3.5 ms, S7 = 165 ± 8.3 ms). The vertical and horizontal lines represent no deviation from the mean perceived time.

To investigate whether real movements could interfere with the perceived time distortions observed here, we asked two additional naïve participants to perform actual hand movements rather than isometric contractions. The psychometric functions for both participants calculated at two critical times with respect to hand movement onset (−0.075 s and −0.025 s) are shown in Fig. 6. At movement onset (−0.025 s, right graphs) perceived time shows opposite effects for the right (relative expansion) and left (relative contraction) directions, whereas earlier during motor preparation (−0.075 s) this difference is either completely cancelled (S6) or strongly reduced (S7). Again this pattern of results is confirmed by the statistical analysis whereas the permutation test yields a significant difference between right and left at −0.025 s for both participants (S6 and S7); at −0.075 s no significant difference is reported for S6 while a significant difference is shown by S7, although this difference is half (23 ms) of the difference observed for the same participant at −0.025 s (43.5 ms), in agreement with the results obtained in the isometric condition.

**Figure 7:**
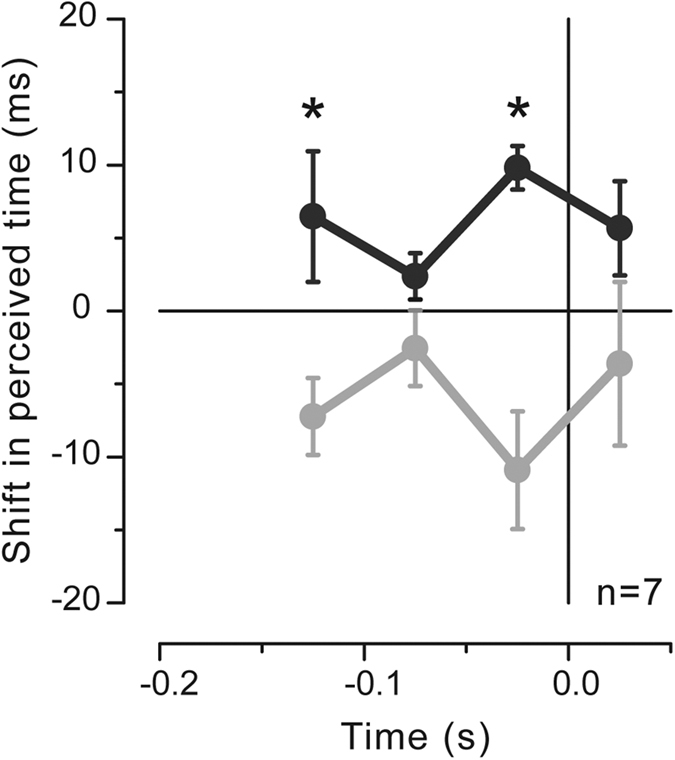
Time course of the shift in perceived time for the right (black) and left (gray) directions averaged across all tested subjects in the two different motor tasks (n = 7; isometric contraction and movement condition). Error bars are standard errors of the mean. Asterisks represent the significance level of post-hoc t-tests (*p < 0.05, **p < 0.01; Bonferroni-corrected for multiple comparisons).

Given the similarity of the time distortion patterns for the isometric and the actual movement conditions, we averaged the PSEs over all participants and the two different motor conditions (n = 7, see Fig. 7). A significant effect of movement direction (F_(1,6)_ = 8.409, p = 0.027) as well as a significant interaction between direction and latency (F_(3,18)_ = 3.25, p = 0.046) and no significant effect of latency (F_(3,18)_ = 0.074, p = 0.973) are reported when analyzing the data from the whole set of participants, proving the robustness of the movement-related temporal modulations. Post-hoc tests show significant differences between perceived time for the right and left directions at −0.125 s (mean difference = 13.7 ± 3 ms; p = 0.024, Bonferroni-corrected) as well as at −0.025 s (mean difference = 20.7 ± 4 ms; p = 0.012, Bonferroni-corrected), but not at −0.075 s (p = 0.258) and at +0.025 s (p = 0.302), indicating that contractions and expansions of time alternate during the motor preparatory stage for rightward and leftward hand movements.

On the contrary, motor preparation for hand movements in both directions does not induce any modulation in the precision of the temporal estimates (p = 0.088 main effect of factor direction; p = 0.545 main effect of factor latency; p = 0.636 interaction effect), confirming that the perceptual biases in visual timing are not systematically associated with a worsening (or an improvement) in temporal discrimination.

## Discussion

It is well-known that eye movements induce dramatic changes in visual perception, including the perception of time^11^,^19^. More surprisingly, a few studies have reported visual temporal distortions even when performing hand/arm movements^13^,^18^, suggesting that the phenomenon may not be related to the inherent connections between the visual and the oculomotor system.

The present study shows alterations in visual time during motor preparation for two different hand movement tasks, providing three important pieces of evidence in favor of a motor-driven account of the observed temporal distortions. First, comparable modulations of visual event-timing are found in isometric and in actual movement conditions, implying that displacement in space of the motor effector and consequent reafferent inputs do not play any causal role in the perceptual effects. Most importantly, apparent time is affected by specific features of the planned movements, namely movement direction. Finally, the effect of movement direction on temporal judgments is not constant, but rather undergoes dynamic modulations in the brief moments preceding movement initiation.

Preparing to move the hand to the right is associated with a general overestimation of time while preparing to move the hand in the opposite, left direction, with a general compression of time. Although small, this directional bias is highly consistent across subjects.

Our findings cannot be due to changes in physical space or movement-induced visual masking, given that the time alteration was observed during an isometric motor task. Nonetheless, the difference in perceived time between rightward and leftward movements could be the consequence of a spatial remapping processes or shifts in spatial attention involved during movement planning. Space and time show strong, reciprocal interactions. When the hands are in a crossed configuration the temporal order of tactile stimuli appears inverted^20^. This temporal bias progressively increases within a 250 ms-window before moving the hands in the crossed posture, suggesting that a prediction of the ensuing body state is built up during motor preparation and is capable of affecting temporal judgments^31^. Time may be encoded not only in body-centered but also in external coordinates. For example, it has been shown that the duration of visual stimuli is underestimated in the left hemispace and overestimated in the right hemispace^32^ as if it was mapped into a spatial representation-a mental left-right oriented line-similarly to what has been suggested for numbers^33^,^34^. Preparing to move to one direction might automatically engage the representation of space associated with the predicted consequence of the upcoming movement, even though no spatial displacement occurs, as in the isometric condition. Moreover, motor preparation for eye as well as for hand movements is known to drive attention towards the target location^35^,^36^,^37^,^38^. Therefore, time could be systematically affected by the direction of the prepared movement just because of its intrinsic spatial coding.

Although the influence of spatial factors, either via predictive or attentional mechanisms, can explain the directional bias for time distortions, spatial factors alone cannot account for the dynamic modulations in perceived time that we observed. The influence of movement direction on temporal judgments is not constant but rather undergoes non-monotonic variations during the motor preparatory period. Expansions and contractions of time alternate in a seemingly cyclic and opposite manner for right and left movements. The fluctuating pattern is confirmed by the significant statistical interaction between movement direction and stimulus latency reported in the isometric contraction condition (n = 5) and for the complete data set that includes also the real movement condition (n = 7). At variance with the TOJ error rates^31^, that change monotonically with time from movement onset for both movement directions, we observed that performance fluctuates and 75 ms before movement onset, corresponding to about half time of the programming phase, perceived time is most similar for the two hand directions. Interestingly inversion of the perceived order of visual events is often observed at the latency of 75 ms before an eye or a hand movement^11^,^19^,^29^, indicating that this may be a crucial interval where time is mostly affected. It is not clear at present how to reconcile these different results. However, they all consistently show a high susceptibility of visual time perception during the programming phase of a body movement.

In conclusion, the reported temporal distortions affect the visual modality, that is a sensory channel which is not directly connected to the hand motor effector. The distortions are found in isometric conditions, excluding movement-related sensory masking and suppression effects^26^,^27^. Interestingly, they are modulated in a non-monotonic fashion by basic motor parameters such as the direction of the movement. Given these properties, attention^39^,^40^ as well as spatial coding and remapping^29^ are unlikely explanations of the phenomenon. Overall, our findings point to a primary contribution of motor processes.

That the motor system might have a leading role in timing functions is in agreement with accumulating neurophysiological evidence showing significant involvement of motor areas in temporal judgments, even in the absence of any overt motor response^41^,^42^,^43^,^44^.

Intriguingly, we recently showed that motor processing and visual processing might be coupled through oscillatory mechanisms^45^. We found periodic fluctuations in visual contrast sensitivity during motor preparation for a voluntary hand movement. Multiple lines of evidence suggest that sensory and motor functions could be regulated by rhythmic processes reflecting alternating states of neuronal excitability^46^,^47^. Our findings support the hypothesis that visual and motor oscillatory activities might become transiently synchronized at the time of action^45^.

By virtue of their periodic nature and their multiplexed frequency-code, oscillations represent a plausible physiological candidate to sub-serve timing functions, especially in the millisecond scale. Brain oscillations have been already proposed as a mechanism to instantiate temporal predictions when sensory information has a regular structure^48^,^49^,^50^ and to explain some illusions in the temporal domain^51^,^52^. Interestingly, the idea that brain rhythmic activity could serve to parse sensory information and order events in time has been already explored a few decades ago^53^,^54^ and corroborated by most recent studies showing that the power^55^, the phase^56^,^57^ as well as the frequency^58^ of brain oscillations affect simultaneity judgments and sensory fusion-thresholds.

The current data do not allow to assess the presence of periodic components in the behavioral time-series because visual temporal judgments were probed within a too short time window preceding movement onset. Yet, the observed alternating pattern is suggestive of underlying rhythmic processes. Curiously, perceived time shows opposite variations during preparation for right and leftward movements, as if the two movement directions were characterized by an anti-phase relationship. The opposite trend in the perception of visual time for right and left movements recalls the anti-phase rhythmicity in visual performance recently shown across different locations in space (and object’s parts)^59^,^60^,^61^; however, we currently lack evidence to tell whether the two phenomena do have some physiological commonalities to be traced in brain oscillatory signals.

The potential involvement of brain oscillatory dynamics in the observed temporal distortions remains at this stage purely speculative. However, it is worth noting that neurophysiological evidence indicates that significant information about movement direction is contained in low-pass filtered MEG and EEG signals^62^, likely corresponding to the readiness potential, as well as in power modulations of low-frequency bands (<13 Hz) in the monkey motor cortex^63^, even before the onset of movement.

Our findings show that motor features of the intended body movements affect perceived visual time in a non-trivial way. A fascinating possibility, to be further explored in future research, is that distortions of time may be the result of more basic changes in visual processing occurring at the time of movement, that might sub-serve fine sensory-motor control mechanisms.

## Additional Information

**How to cite this article**: Tomassini, A. and Morrone, M. C. Perceived visual time depends on motor preparation and direction of hand movements. *Sci. Rep.* **6**, 27947; doi: 10.1038/srep27947 (2016).
